# Stabilization of Microrobot Motion Characteristics in Liquid Media

**DOI:** 10.3390/mi9070363

**Published:** 2018-07-23

**Authors:** Ali Anil Demircali, Huseyin Uvet

**Affiliations:** Department of Mechatronics Engineering, Yildiz Technical University, 34349 Istanbul, Turkey; aanil@yildiz.edu.tr

**Keywords:** microrobots, control systems, untethered manipulation, diamagnetic levitation

## Abstract

Magnetically actuated microrobot in a liquid media is faced with the problem of head-tilting reaction caused by its hydrodynamic structure and its speed while moving horizontally. When the instance microrobot starts a lateral motion, the drag force acting on it increases. Thus, the microrobot is unable to move parallel to the surface due to the existence of drag force that cannot be neglected, particularly at high speeds such as >5 mm/s. The effect of it scales exponentially at different speeds and the head-tilting angle of the microrobot changes relative to the reference surface. To the best of our knowledge, there is no prior study on this problem, and no solution has been proposed so far. In this study, we developed and experimented with 3 control models to stabilize microrobot motion characteristics in liquid media to achieve accurate lateral locomotion. The microrobot moves in an untethered manner, and its localization is carried out by a neodymium magnet (grade N48) placed inside its polymer body. This permanent magnet is called a carrier-magnet. The fabricated microrobot is levitated diamagnetically using a pyrolytic graphite placed under it and an external permanent magnet, called a lifter-magnet (grade N48), aligned above it. The lifter-magnet is attached to a servo motor mechanism which can control carrier-magnet orientation along with roll and pitch axes. Controlling the angle of this servo motor, together with the lifter-magnet, allowed us to cope with the head-tilting reaction instantly. Based on the finite element method (FEM), analyses that were designed according to this experimental setup, the equations giving the relation of microrobot speed with servo motor angle along with the microrobot head-tilting angle with servo motor angle, were derived. The control inputs were obtained by COMSOL^®^ (version 5.3, COMSOL Inc., Stockholm, Sweden). Using these derived equations, the rule-based model, laser model, and hybrid model techniques were proposed in this study to decrease the head-tilting angle. Motion control algorithms were applied in di-ionized water medium. According to the results for these 3 control strategies, at higher speeds (>5 mm/s) and 5 mm horizontal motion trajectory, the average head-tilting angle was reduced to 2.7° with the ruled-based model, 1.1° with the laser model, and 0.7° with the hybrid model.

## 1. Introduction

Microrobot actuation studies have focused on electromagnetic methodologies associated with different control approaches. Their locomotion techniques have a crucial role for some major areas such as invasive diagnostics and targeted drug and living cell delivery. Due to the non-linear nature of the magnetic field, the precise localization of microrobots in the in-vitro/vivo application is becoming a challenging field for researchers [1]. Promising solutions can be developed by implementing not only material-based solutions, but also a combination of sensors and control models. Miniaturization strategies of existing robotic technologies and biomimetic approaches make effort towards moving microrobots in a predefined trajectory in different media [2,3]. For example, a levitated microrobot in liquid media cannot move parallel to the surface during horizontal movements due to their hydrodynamic structures. We named this problem the “head-tilting” reaction. Previous studies were applied to various levitation techniques for the stabilization and control of the horizontal movements of microrobots [2,3,4,5]. Based on these studies, active levitation techniques that use an electromagnet give better results compared to passive levitation techniques that use permanent magnets. However, an application of active levitation requires expensive and complex feedback mechanisms, and has a higher energy consumption rate. Passive levitation is generally more favorable, since it is more compact, consumes less energy, and can be applied at room temperature [6,7,8]. Furthermore, it is observed that open loop control methods yield good results when applied to passively levitated microrobots. It is possible to obtain satisfactory results in microrobot studies by using various control methods such as single degree-of-freedom models [9,10]. With these methods, even with simple control strategies, good results can be observed. Moreover, there exist previous studies in which passive levitation was applied to living cells and water bubbles to levitate them to a target position [11,12,13]. In another study, Perline and his team applied open-loop controllers to colonies of diamagnetically levitated microrobots [8]. It can be seen that high-accuracy motion control is crucial for many applications, such as tissue engineering, cell manipulation, drug delivery, microassembly, and protein-crystal handling [14,15,16,17,18,19,20,21,22,23,24,25,26,27].

When working with micro-scale robots, the fluidic environment shows laminar flow characteristics; therefore, the Reynolds Number of the flow cannot be neglected. Nelson and his team investigated the effects of drag force during electromagnetic levitation [28]. However, they could not get adequate results in the control of the robot due to lack of modelling of environmental effects. Similarly, Sitti and his team had difficulties controlling microrobots due to lack of theoretical calculation of net force [29]. Assumptions made about the drag force hindered their accuracy of motion. Khamesee and his team obtained similar results, even though they added drag force into their control model [5]. Arai and his team applied high-frequency ultrasound vibrations on the surface upon which the microrobot moves and consequently achieved better results [30]. However, the drag force on the microrobot still exists and their motion control strategy does not answer it. Feng Lin and his team manufactured a microrobot using pyrolytic graphite and a diamagnetic material and levitated it using an electromagnet with four poles [31]. However, their motion range was limited to 1 mm, and no accuracy of motion was studied. Also, since the study does not contain any mathematical model of the motion characteristics, the deviation of experimental results from the theoretical calculations cannot be seen.

In our study, we control the lateral motions of an untethered microrobot via passive diamagnetic levitation in a fluidic environment. A disc-shaped magnet (N48 grade neodymium) with ø1 mm and 200 µm thickness, called the “carrier-magnet”, is used at the center of a microrobot and it is also placed above on a pyrolytic graphite surface in a liquid medium. A ring-shape magnet (N48 grade neodymium) with dimension of ø40 mm × ø20 mm × 8 mm, called the “lifter-magnet”, is attached on a micro-stage and aligned with the microrobot in order to provide stable magnetic levitation. This setup, compared to others, is more compact and requires less energy to operate than actively controlled magnetic levitation systems. While the microrobot motion trajectory is on the horizontal axis, it cannot accelerate parallel to the pyrolytic graphite surface. The head-tilting reaction starts and develops depending on the horizontal speed of the microrobot and increases exponentially in time. Furthermore, the drag force also increases exponentially as a function of microrobot’s speed, and it cannot be omitted. Therefore, before determining control parameters, the speed, mechanical delay between permanent magnets, and orientation of carrier-magnet were modelled in COMSOL^®^ (version 5.3, COMSOL Inc., Stockholm, Sweden). Afterwards, control strategies were applied to minimize the drag force’s effect to realize stable locomotion characteristics even at a much higher speed (>5 mm/s). Experimental results show that the microrobot may successfully have followed a sinusoidal trajectory of 150 µm amplitude and 4 mm period with an average error of 1.73 µm.

Our contribution to this study is to design 3 control models that take hydrodynamic effects exerted on the microrobot into account. Also, for the first time, the problem of head-tilting reaction is investigated and solutions are presented based on these three control models, which are the rule-based model, the laser model, and the hybrid model. The main purpose of the developed control techniques is the minimization of the drag force’s effect on the microrobot, which moves inside a fluidic environment in an untethered manner. Thus, they can be utilized in any application that requires complex trajectories tracking and precise transportation of micro and nano structures. The rule-based model uses a constant angle, which is calculated via projected duration of motion and motion speed, as input parameters. For the laser model, the control input frequency changes between 15 (66.7 ms) Hz and 58 (17.2 ms) Hz. On the other hand, the hybrid model takes advantage of those two previous strategies and combines them into a single model. In the experimental results section, the reliability of these three models is verified, and the results are presented. All control techniques are compared to the uncontrolled head-tilting reaction.

## 2. Mathematical Model

**Figure 1:**
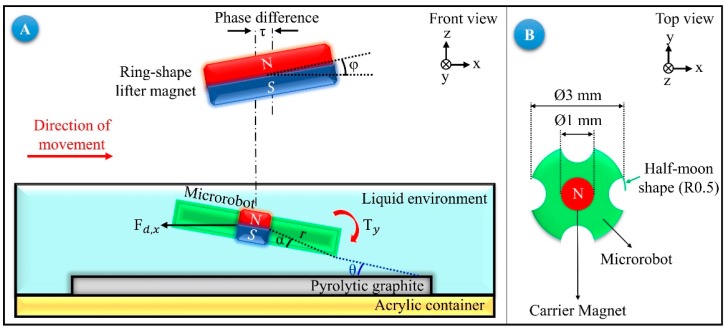
(**A**) The head-tilting reaction is introduced in the uncontrolled state of the microrobot moving in the X direction. (**B**) Microrobot whose dimensions are: ø3 mm × ø1 mm × 0.2 mm, shown from top view.

In this section, we explain the mathematical model that can be used for calculating the minimum and maximum levitation heights of the microrobot, the microrobot’s speed-phase difference of the stage, and its head-tilting angle equations. In our experiments, we observed that the horizontal motion of the microrobot causes a head-tilting reaction; consequently, it is unable to move parallel to the surface. Two main reasons were concluded for this situation. These reasons are the “phase difference” between the center of the microrobot and the center of the carrier-magnet, and the drag force that acts on the robot due to its hydrodynamic structure. The phase difference occurs because of the fact that the acceleration of microrobot is lower than the carrier-magnet. This difference in acceleration is caused by the air resistance acting only on the lifter-magnet, which is connected to the micro-stages during its movement. While the resistance of air is negligible, the drag force acting on the microrobot in the fluid cannot be omitted. For this reason, while the lifter-magnet follows a step function like a motion profile with sharp edges, and the microrobot has a parabolic motion profile with smoother edges [32]. Figure 1 shows the schematic diagram of the head-tilting reaction and phase difference for the microrobot moving in the *x* axis. In this figure, a pyrolytic graphite is placed on the surface of an acrylic container. The microrobot is located above the pyrolytic graphite in liquid media. For our model and further experiments, the liquid media is chosen as de-ionized (DI) water. Since the pyrolytic graphite is a diamagnetic material with a magnetic permeability of $\mu_{r}=0.999992$, it encloses the microrobot within the boundaries of magnetic field lines of the lifter-magnet. Because of this, we can achieve more stable levitation characteristics inside the liquid [32,33].

During this movement, an undesired torque $T_{y}$ in the *y* axis is exerted on the microrobot, which causes it to tilt in the direction of motion. Also, a phase difference, *τ*, is observed, which is caused by the aforementioned reasons. The lifter-magnet’s position is controlled in three-axes using microcarrier stages, and its orientation is controlled in two axes by using two servo motors. The orientation angle of the lifter-magnet in the direction of motion is expressed by $\varphi_{y}$. The microrobot radius is shown by *r*, the angle between the corner point and the center by *α*, the angle between the corner point and the pyrolytic graphite by *θ*, and the drag force acting on the movement in the x axis direction by $F_{d,x}$.

**Figure 2:**
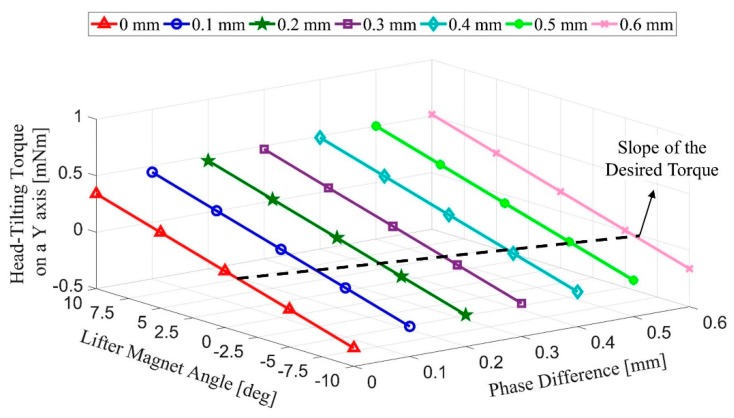
The angle calculation to be applied to the lifter-magnet is presented to eliminate undesired torque.

As shown in Figure 1, the microrobot moving in the x direction is not parallel to the surface during its motion due to the undesired torque $T_{y}$ acting on it. For a microrobot, for which the moment of inertia is taken as I=23.62 (μg mm^2^), a relation between the angular acceleration and undesired torque can be determined [32,33,34]. Accordingly, Equation (1) will be used to calculate the angle value at which the lifter-magnet should be held in order to avoid the generation of undesired torque values (Figure 2).

(1) $$\ddot{\theta }=\frac{T_{y}+\left(-F_{D,x}+F_{m,x}+F_{g,x}\right)sin\left(\theta \right)r}{I}$$

**Figure 3:**
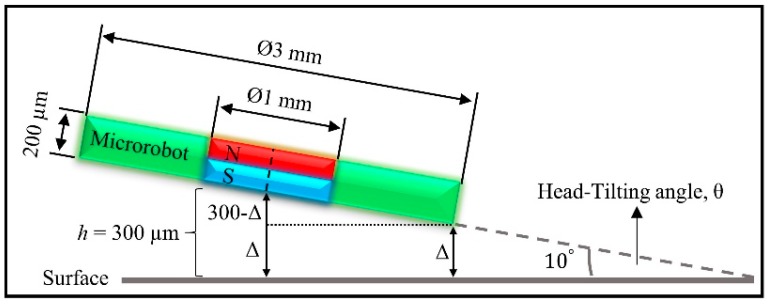
Illustration of levitation height determination in relation to the microrobot-head-tilting angle.

The head-tilting angle, *θ*, increases with the speed of motion, and this has some implications for robot levitation. Increasing head-tilting angles can cause the microrobot to hit the surface during its motion. In Figure 3, the head-tilting amount, Δ, can be calculated for a robot with *α* = 3.814°, *θ* = 10°, h=300 μm, and *r* = 1503.33 μm

(2) Δ=h−rcos(α)sin(θ)

**Figure 4:**
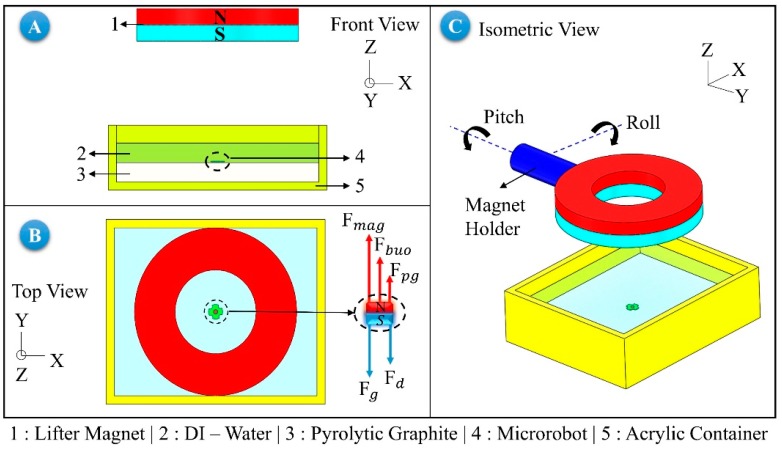
Diagram of magnetic levitation system used in experiments. Numbers represent part names. (**A**) shows vertical alignment of microrobot and lifter-magnet; (**B**) shows forces exerted on microrobot, which is aligned with lifter-magnet; and (**C**) illustrates isometric view of the whole experimental setup.

Δ = 39.528 μm is obtained for h=300 μm, which is the instantaneous levitation height. According to this calculation, if the angle of diversion of the microrobot is 10°, and if the levitation height is lower than 260 μm, it is determined that the robot is likely to be hit the surface. If the instantaneous levitation height is lower than the required tolerance between the microrobot and the surface, the robot may hit the surface. For this reason, the working boundaries of the microrobot should firstly be determined. To find these boundaries, a free-body diagram needs to be generated, and each force acting on the robot needs to be calculated. Figure 4A shows the front view of the levitation system. The top view of the system and the forces acting on the robot are shown at Figure 4B, and the isometric view of the lifter-magnet and the test setup is shown in Figure 4C. In Figure 4B, the magnetic force of the lifter-magnet acting on the microrobot is illustrated as $F_{mag}$; the buoyant force due to liquid media as $F_{buo}$; the diamagnetic force induced by the pyrolytic graphite, which is located on the surface of the acrylic container (shown in Figure 1A and Figure 4A), as $F_{pg}$; the gravitational force as $F_{g};$ and the drag force as $F_{d}$.

The acceleration-dependent mathematical model is expressed in Equations (3)–(5), in which the robot mass is *m_r_* [32,33,34]. The forces exerted on the microrobot are shown in Figure 4B. For a robot with a mass of 2.92 μg, robot acceleration can be determined for known values of lifting force (12.788 μN), speed-dependent friction force [33], and gravitational force (28.741 μN). The relationship between phase difference and the microrobot acceleration has been investigated in a different study [32].

(3) $$\ddot{x}=F_{d,x}+\frac{\left(F_{mag,x}+F_{pg,x}\right)}{m_{r}}$$

(4) $$\ddot{y}=F_{d,y}+\frac{\left(F_{mag,y}+F_{pg,y}\right)}{m_{r}}$$

(5) $$\ddot{z}=F_{d,z}+F_{buo,z}-F_{g,z}+\frac{\left(F_{mag,z}+F_{pg,z}\right)}{m_{r}}$$

Using a mathematical model with 3 degrees of freedom, which takes into account gravity, lift, friction, and magnetic and diamagnetic forces, it is calculated that the net magnetic force should be 16.74 μN in order to levitate the microrobot [32,33,34]. Applying these forces on the microrobot causes it to levitate in the fluidic environment. It is necessary to calculate the robot and lifter-magnet accelerations so that the approximate values for phase difference can be found during the lateral motion of the robot. The aim of the experiments was to control microrobot motion in high accuracy so that it could complete a predetermined trajectory in a repeatable manner at desired speed. Also, how fast this task can be accomplished is another important criteria. Since the lifter-magnet speed profile is determined (step function), the value of the acceleration can also be found. However, the acceleration of the microrobot depends on the speed of the lifter-magnet. For example, although the lifter-magnet completes a trajectory of 5 mm in 1 s with a speed of 5 mm/s, it takes time for the microrobot to reach a speed of 5 mm/s due to existence of the drag force. It has been shown that the value of $F_{d,x}$ is 0.125 μN for 5 mm/s speed [33]. For a microrobot with a constant mass, it has been stated that the speed profile, and thus the acceleration, may be of parabolic increasing-decreasing characteristic against the variable drag force [32]. The value of the microrobot acceleration can be determined according to Equation (6).

(6) $$F_{mag,x}-F_{d,x}=m_{r}a_{r}$$

For the microrobot whose acceleration is determined, instantaneous speed values can be found by assuming that it has an acceleration characteristic. Using microrobot speed rating, $V_{r}$, Equation (7) can be used to calculate the time required to reach a speed of 5 mm/s.

(7) $$v_{r}=v_{0}+\int_{0}^{t}adt$$

The drag force, which is equal to about 1/134 of the net magnetic force [35], causes the microrobot to have a delay of 103.1 ms in total, because the starting values are 7.46 ms and 5 mm/s. As a result, according to the speed value, it is expected that the robot will move with *τ* = 515.5 μm. In addition, a linear expression can be obtained from previous works done with the phase difference, and approximate phase difference expression can be related to the speed by Equation (8) [32,33].

(8) $$\tau =0.134v_{r}-0.146$$

## 3. Simulation Studies

**Figure 5:**
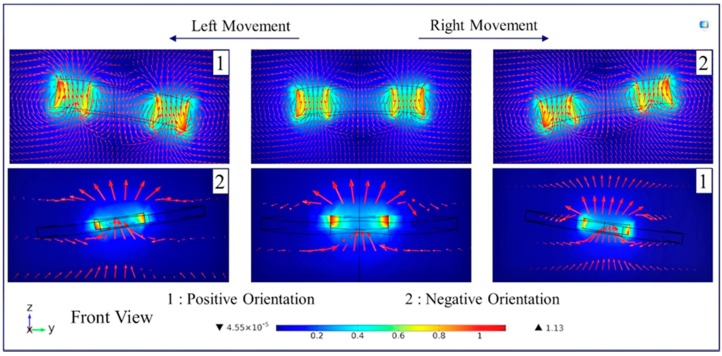
Analysis to find the orientation of the carrier-magnet in order to avoid head-tilting reaction. Accordingly, it is determined that the carrier-magnet must have a direction opposite to the motion direction of the microrobot.

In the mathematical model section, it was shown that the robot head can hit the surface if the levitation height is not sufficient, and the definitions for head-tilting and phase difference were stated. Also, a method for determining acceleration of microrobot in order to find the phase difference was given. In this section, the simulation studies are presented, which were conducted in order to develop a method for improving the head-tilting action that acts during the lateral movements of the microrobot. Using the simulation results, we also obtained the following microrobot control parameters via orientation equations, the operation limits, and the expression for surface impact condition. In addition to them, a first-order linear equation for the phase difference is calculated. The aim is to perform the robot motion parallel to the surface as shown in Figure 4C. In the analyses made on the COMSOL^®^ AC/DC module, the orientation that must be applied on the lifter-magnet should firstly be determined. In Figure 5, the magnetic field force lines that pass through the lifter-magnet and microrobot surface are shown when they are parallel to the surface. It has been determined that the field lines on the robot with levitation on the *z* axis are perpendicular to the robot. A parametric analysis on the tilting angle of the microrobot is performed in order to determine the direction of the force applied during right and left movements. As a result of the analysis, it was found that the robot and the lifter-magnet must have opposite orientations. In this case, the active force lines on the robot were observed to remain perpendicular to its surface.

Microrobots have a small characteristic length *L* and a small characteristic velocity $V_{s}$; this leads to a small Reynolds (*Re*) number (Equation (9)) and Stokes flow (Equation (10)) [35].

(9) $$Re=\frac{\rho_{f}V_{s}L}{\mu }\ll 1$$

**Figure 6:**
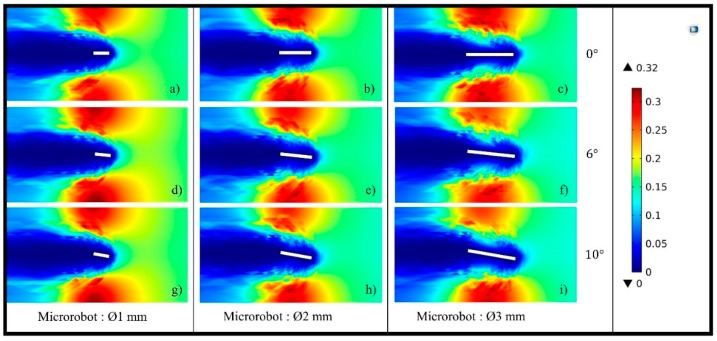
Calculation of *Re* number is simulated at different head-tilting angles (0° (**a**,**b**,**c**), 6° (**d**,**e**,**f**) and 10° (**g**,**h**,**i**)) for microrobots with same thickness (200 μm) and different sizes (1 mm (a,d,g), 2 mm (b,e,h), and 3 mm (c,f,i)). COMSOL Creeping Flow analysis is utilized in stationary liquid media. The legend on the right side, as expected, shows that the *Re* ≪ 1 is not dependent on size and head-tilting angle.

At low *Re*, the Navier-Stokes Equation becomes time-independent [36],

(10) $$\rho \left(\frac{\partial v}{\partial t}+\left(v.\nabla \right)v\right)=-\nabla p+\mu \nabla^{2}v$$

in which v is the velocity vector field and p is the hydrodynamic pressure scalar field. A low *Re* number can be due to a slower motion, a small working environment, or high viscosity [37]. Navier can be omitted, since Re≪1 [38] and inertial terms of fluid become negligible, while viscous force and surface tension are more dominant at the small length scale. In the studies with a low *Re* regime, it can be seen that size effects can be neglected, and locomotion of a microrobot can be achieved by vibrating it. Depending on the vibration frequency, the p value is changed, and motion under low *Re* can be obtained [35,36,37,38]. In our work, velocity of the microrobot determines the flow characteristic, since DI water remains stationary in the container. Thus, the motion characteristics of the microrobot in a low *Re* flow regime is investigated depending on its scale and with a head-tilting angle, which varies between 0–10° (Figure 6). Creeping Flow Physics in COMSOL^®^ is used for three different sizes of microrobot (1 mm, 2 mm, and 3 mm). In this case, when the experimental conditions are kept constant, the change in the Reynolds number is dependent on the speed of the robot, not its size and head-tilting angle. In addition, for microrobots of different sizes with the same flow characteristics, the drag force does not change in the movements parallel to the surface [32,33,34]. However, for microrobots that are working in the same experimental conditions, have the same speed, same head-tilting angle and different sizes, the drag force will change proportionally with the cross-sectional area. From Equation (1), the torque value that should be applied to the microrobot can also be calculated.

**Figure 7:**
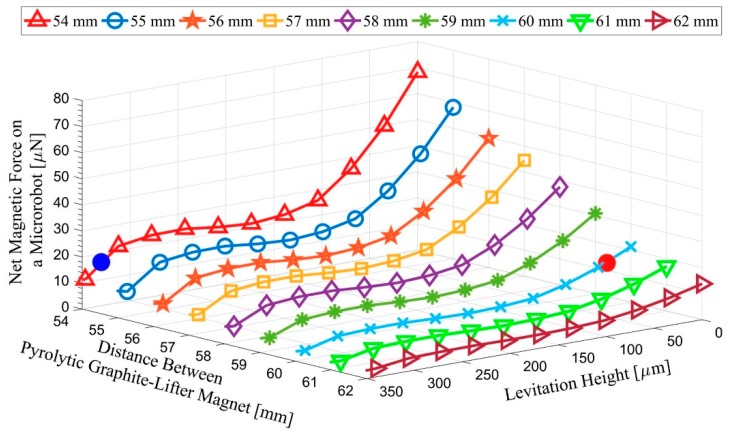
Microrobot study limits are determined. Accordingly, the minimum and maximum points are shown in the range of 31.0 μm (red)–329.1 μm (blue).

Once the lifter-magnet orientation is found, it is also very important to determine the operation limits of the microrobot. As indicated in the mathematical model section, the robot has a risk of surface collision, depending on its levitation height and speed. There is a risk of the microrobot surfacing in an uncontrolled manner due to close proximity to the lifter-magnet. These situations can be addressed by setting a lower and an upper limit to the levitation height. Here, Figure 7 represents a graph for the net force exerted on the microrobot against microrobot lifter-magnet distance at different levitation heights. According to the net magnetic force value of 16.74 μN [32,33,34], the microrobot was observed to be at the maximum levitation height of 329.1 μm when the lifter-magnet was at a distance of 54 mm (marked with a blue dot) from the pyrolytic graphite. On the other hand, the microrobot was at the minimum 31.0 μm levitation height when the lifter-magnet was located at 60 mm height (marked with a red dot). Since the magnetic force exhibits an exponential characteristic, the linear region of 100–300 μm was chosen as the operation limits. In particular, this region is affected less by the nonlinear magnitudes for experimental speed ranges.

**Figure 8:**
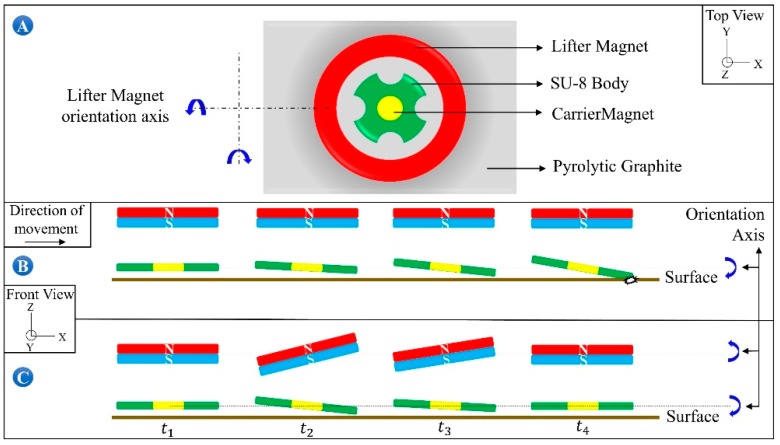
Roll and pitch axes control the orientation of the microrobot to eliminate head-tilting reaction (**A**); result of the uncontrolled microrobot motion is given in (**B**), and the head-tilting reaction can be eliminated by adjusting carrier-magnet orientation (**C**).

Following the determination of operation limits, the head-tilting reaction after certain time intervals is examined, and results are presented. By the time the microrobot reaches a desired speed with a certain delay parameter due to a lower acceleration, the tilting angle is also found to be dependent on this parameter. Figure 8A shows the top view of the system and the orientation of the lifter-magnet. Figure 8B shows a depiction of the microrobot that is starting to move by tilting its head gradually in the x direction at *t*_1_ until it reaches the desired speed at *t*_4_. At *t*_4_, the microrobot is depicted as hitting the surface due to its higher speed. Moreover, the simulation data presented in Figure 8C shows that the lifter-magnet is given an opposite orientation relative to microrobot. It is observed that the head-tilting angle of the microrobot is reduced starting from time *t*_2_. At time *t*_4_, instantaneous speeds are equal and the motion characteristic is depicted as being parallel to the surface.

For all cases mentioned in the mathematical model section, finite element method (FEM) studies were conducted, and their results are given in this section. After the determination of these results, we need to derive the equations necessary for rule-based model design. To this end, various tilting angle values of microrobot were studied in COMSOL^®^ by parametric analysis. Different phase difference values are presented relative to the *x*-axis with the upper legend in Figure 8, while different angles of microrobot are given on the *y*-axis. On the *z*-axis, undesired torque values for different phase differences and carrier-magnet angles are shown. In this graph, a black dashed line is used to show the values where the torque equals zero. This line shows the angle that must be applied to the lifter-magnet in order to overcome the undesired torque.

Equations (11) and (12) are derived using the dashed line shown in Figure 8. These equations are related to the speed of the micro-stage and its tilting angle to be applied to the rule-based model and the laser model, respectively.

(11) θ=1.323v−0.529

(12) θ=1.8761φ+0.541

As a result of using these equations with a certain time delay depending on the speed, the head-tilting angle can be improved, as presented with the results in experimental study section.

## 4. Experimental Study

In this section, the experimental studies were presented in accordance with the simulation results, which were grounded on the mathematical models obtained in Section 2. In the light of simulations and mathematical models, the lower and upper limits of magnetic levitation are determined, and the control algorithms are created. Afterwards, microrobot is tested for different scenarios such as following a complex sinusoidal trajectory and longitudinal motion control at a constant levitation height, and results are then compared.

### 4.1. Experimental Setup

**Figure 9:**
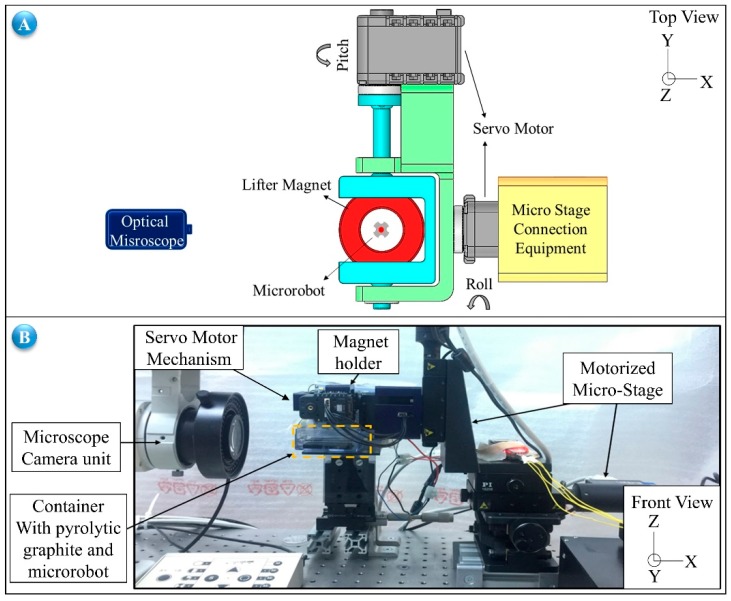
(**A**) Illustrates the experimental system with 2 servo motors, micro-stage connection apparatus, and lifter-magnet microrobot alignment. In (**B**) the full testing and measurement systems are shown.

Based on the derived mathematical model and conducted simulations, it is found that the microrobot can be levitated in the range of 31.0–329.1 μm, and the undesired torque on the robot can be overcome when the orientation of the lifter-magnet is opposite to that of microrobot in the direction of motion. Also, the approximate linear equation of the phase difference is given as a function of the horizontal speed of the microrobot in Equation (8). In this section, the theoretical and simulation results are compared with the experimental results, and the experiments for each control algorithms are explained. Pyrolytic graphite is placed on the surface of an acrylic container filled with deionized water (DI-water). The lifter-magnet is placed on top of the microrobot, which consists of an N48 neodymium magnet and an SU-8 frame. The component that connects the carrier-magnet to the micro-stage is printed in a 3D printer. This component is designed to enable controlling of the orientation of the microrobot using two servo motors (Figure 9A). An optical microscope (Olympus SZX-7, Olympus Corporation, Tokyo, Japan and PointGrey GS3-U3, FLIR Integrated Imaging Solutions Inc., Richmond, BC, Canada) is used for imaging the experiment (Figure 9B). On the *z* axis, the head-tilting angle and levitation height were measured by a sub-micrometer-resolution laser sensor (optoNCDT-ILD2300-50, Micro-Epsilon, Raleigh, NC, USA).

**Figure 10:**
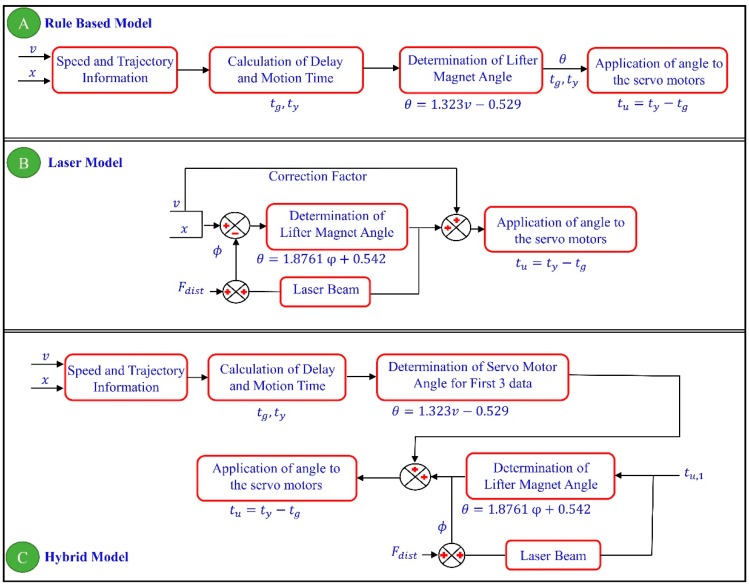
(**A**) Shows the rule-based model (open-loop), (**B**) shows the laser model (closed-loop), and (**C**) shows the hybrid model control block diagrams.

With the experimental setup shown in Figure 9, it is possible to control the microrobot with 5 degrees of freedom (5 DOF). In all systems in which a microrobot is intended to be levitated in a fluidic environment using a lifter-magnet, it is inevitable for a phase difference to occur due to the hydrodynamic structure of the system. For this reason, the first control problem we worked on is orienting the lifter-magnet at the optimal angle depending on the horizontal speed of the microrobot such that it will move parallel to the surface (Figure 10A). The inputs of the control system are determined as micro-stage speed (mm/s) and length of trajectory (mm). The motion completion time, *t_y_*, can be calculated easily for a given trajectory using its length and microrobot speed. However, orientation control of the lifter-magnet should not be applied until the microrobot reaches a desired speed so that an undesired torque is not induced on the microrobot. This requirement is explained in a previous study in detail [32]. For this reason, orientation control of the lifter-magnet is applied after a certain time delay, *t_g_*, when the microrobot reaches the desired speed. Before the trajectory is completed, the lifter-magnet should return to the parallel position by reducing the angle as shown in time periods *t*_3_, *t*_4_ in Figure 8B. This ensures that the robot will remain parallel to the surface at the start and at the end. By controlling the lifter-magnet angle according to Equation (11), during the time period *t_u_* as shown in Figure 10, head-tilting angle of the microrobot can be reduced. The second control problem we work on is reducing the head-tilting angle using a closed loop control algorithm (Figure 10B). This time, the head-tilting angle of the microrobot is measured with a laser sensor and Equation (12) is applied. A feed-forward approach is also added to improve the transient response, in accordance with the horizontal speed of the microrobot. The hybrid model is used in the last controller structure. In this model, development of a better control structure is aimed for, which could achieve lower tilting rates and a more stable motion by combining the advantages of the rule-based model and the laser model shown in Figure 10A,B. Initially, the rule-based model shown in Figure 10A is applied in order to improve the transient response. Then, the laser model shown in Figure 10B is used in order to eliminate the steady-state error. The $t_{u,1}$ value shown in Figure 10C represents the elapsed time after the first three angle commands are applied.

It is possible to obtain displacement measurements at the sub-micron level using the OptoNCDT-ILD2300-50 laser sensor. However, it needs to be calibrated first in order for it to be able to measure the head-tilting angle and the levitation height inside the fluidic container, because the laser beam emitted from the laser sensor will exhibit different characteristics inside the fluid and in the air. If the laser beam does not enter into the fluid in a perfectly perpendicular manner, it will diffract, and the accuracy of measurements will lower. For this reason, blocks with different sizes, manufactured in accordance with the DIN EN ISO 3650 standard, are used to calibrate the laser sensor. Different blocks that have different increasing heights of 1 µm, 10 µm, and 100 µm, starting from 0.5 mm, are used. The error rates obtained from the measurement of these blocks are later incorporated into the control structure as $F_{dist}$ (Figure 10B,C).

### 4.2. Robot Levitation

**Figure 11:**
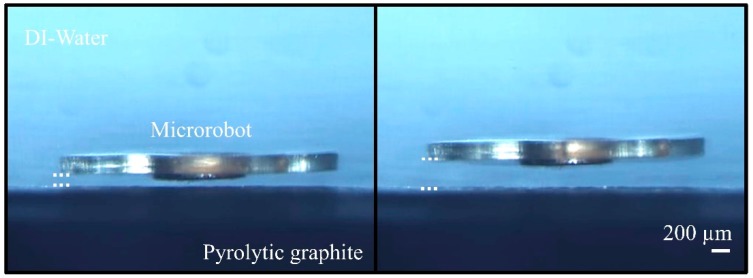
The left picture shows minimum levitation height of the microrobot (30.0 μm) and maximum levitation height (333.8 μm) on the right picture.

Robot levitation is preferred, especially for applications in which very high precision (nano-level) is required. The operation range of the microrobot is affected by various parameters such as the density of the media, temperature and so on. According to the simulations and mathematical calculations made, the operating limits of the microrobot are found to be minimum 31.0 μm (by analysis)–30.0 μm (by experiments) and maximum 329.1 μm (by analysis)–333.8 μm (by experiments) [32,33,34], as shown in Figure 11. The difference in analysis and experiments is caused by the homogeneity of the neodymium magnets used, and the uncertainty in the magnetic flux densities within the given range.

### 4.3. Robot Orientation Capabilities

**Figure 12:**
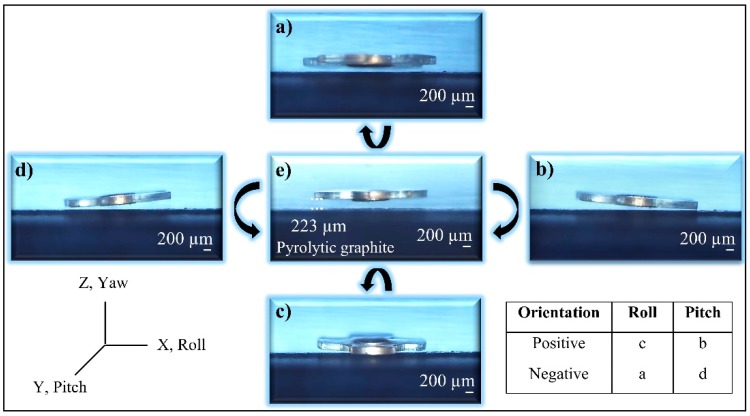
Axial orientations of the robot are shown. (**e**) is the initial position at 223 μm levitation height, (**a**,**c**) shows negative and positive roll angle respectively, and (**b**,**d**) shows positive and negative pitch angle, respectively.

Orientation of the microrobot is controlled by changing the orientation of the lifter-magnet using the 2 attached servo motors (Figure 12). Its lateral movements and control are enabled through the positioning of the lifter-magnet. Angular motions, such as rolls and pitches, are also taken into account to control the orientation of the lifter-magnet with high precision. The greatest advantage of high-precision orientation ability is that the robot can avoid the increase in head-tilting reaction that occurs due to the drag forces during its lateral motion. Figure 12a presents the negative roll, Figure 12b the positive pitch, Figure 12c the positive roll, and Figure 12d the negative pitch orientations. Correspondingly, Figure 12e indicates the stable positioning of the microrobot at certain levitation height (223 µm) parallel to the surface.

**Figure 13:**
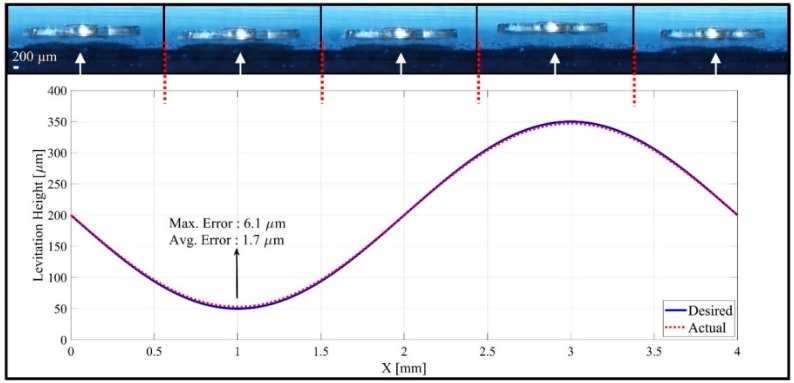
The experimental data represents microrobot motion on a 4 mm trajectory with a sine-wave profile relative to the *x*-axis by the time recording changes on levitation height relative to the *z*-axis. Five snapshots are given to show actual location.

After successfully performing magnetic levitation and 2-axis orientation control of the microrobot, the sine-wave-shaped trajectory is followed to demonstrate the motion capabilities of the microrobot. For the first time, a motion profile with a variable levitation height is applied, and the results are given in Figure 13. With an initial levitation height of 200 μm, the robot is able to follow a trajectory of 4 mm horizontal length and 150 μm amplitude at a speed of 5 mm/s with an average error of 0.536%. It is observed that this margin of error is due to the need for sharp turns at minimum and maximum levitation heights. To calculate testing errors, microrobot motion is recorded at 100 fps, and the levitation height is calculated. An optical microscope unit, which has a wide focal area, has a fixed position such that it records the lateral localization at given frame rate. Additionally, the proximity profiles acquired from the laser sensor are compared to determine maximum and average errors.

### 4.4. Uncontrolled Model and Rule-Based Model

**Figure 14:**
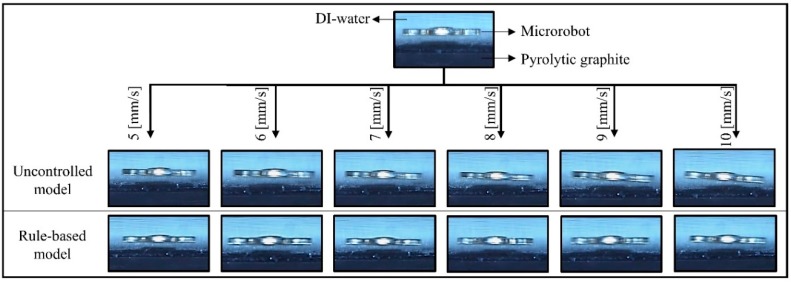
Experimental results to show microrobot head-tilting reaction during implementation of uncontrolled and rule-based models at variable speeds (from 5 mm/s to 10 mm/s). While head-tilting reaction is obvious, in the case of the rule-based model, the microrobot’s lateral motion is slightly developed.

Uncertainties due to laser calibration, the sampling processes and the deviations observed during the control of the microrobot led us to design a controller that would make the system more stable and reduce the errors while following the desired trajectories. The rule-based model, which is an open-loop controller, is ideal for correcting the tilting angles during lateral movements because of the non-linearities that are influential on these effects. As shown in Figure 14, the rule-based model is applied to reduce the head-tilting angle, and it corrected up to a few degrees, depending on the microrobot’s speed (Table 1).

However, steady-state error is observed in the rule-based model due to the absence of a sensory feedback mechanism. Moreover, to improve robustness of the control structure, we decided to develop closed-loop control models, which are the laser and hybrid models.

### 4.5. Laser Model and Hybrid Model

**Figure 15:**
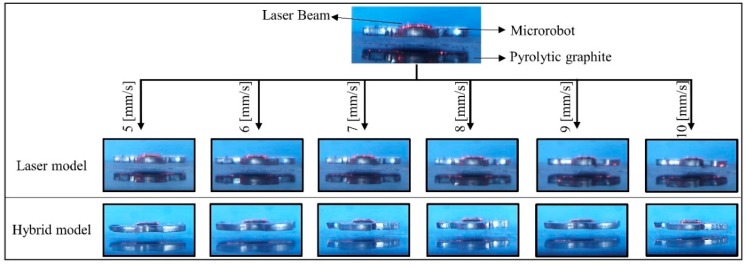
The images taken by the experimental data of the laser feedback and the hybrid model controllers at six different speeds in the range of 5–10 mm/s. In order to make the laser marker visible, the experimental images of the microrobot are presented in isometric view.

Following the open-loop control model used to improve the transient response, the closed-loop models, which are the laser and hybrid models, are tested to decrease steady-state error (Figure 15). The position and orientation information of the microrobot is obtained from a laser sensor placed on top of the experimental setup. The head-tilting angle is calculated from the slope of the line that intersects the lower corner points of the microrobot. It is observed that the transient response of the microrobot can be developed with the laser model, which uses sensory feedback from a laser sensor. The scanning frequency of this sensor varies between 15 Hz and 58 Hz, due to the laser sensor being sensitive to hardware limitations and other dynamic external factors such as ambient light conditions or low-frequency vibrations. The microrobot follows the lifter-magnet with a minimum delay of around 100 ms. Thus, the frequency of the controller needs to be at least 20 Hz. The transient response of the head-tilting angle does not satisfy this requirement at high speeds. Therefore, the laser model is not robust enough to correct the head-tilting angle at higher speeds (above 15 mm/s). For this reason, a hybrid model is developed in order to make a more robust controller to adapt to different environmental conditions and in order to have a speed-independent model (Figures 18 and 19). In this model, the transient response is improved by adapting the rule-based model to the laser model. As a result, the control system can reduce steady-state errors and can be used independently of microrobot speed.

**Figure 16:**
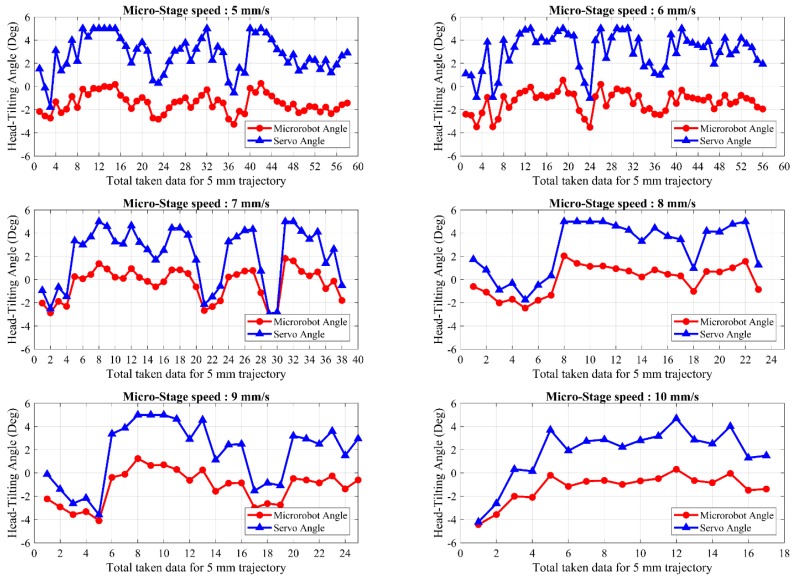
The microrobot head-tilting angle and the applied servo motor angle for the application of the laser model within a speed range of 5–10 mm/s.

Head-tilting angles of the microrobot and the lifter-magnet are shown in Figure 16 in a speed range of 5–10 mm/s when the laser model is used. Orientation control of the microrobot with only laser feedback causes it to exhibit a rolling motion due to the drag force exerted on it along the *z*-axis. Thus, the steady-state error cannot be eliminated. By using only laser feedback, it can be observed in Figure 16 that the head-tilting reaction sometimes surpasses the servo angle and causes inconsistencies in the number of samples and results. For this reason, directing the microrobot using only laser feedback cannot be regarded as a reliable control technique.

**Figure 17:**
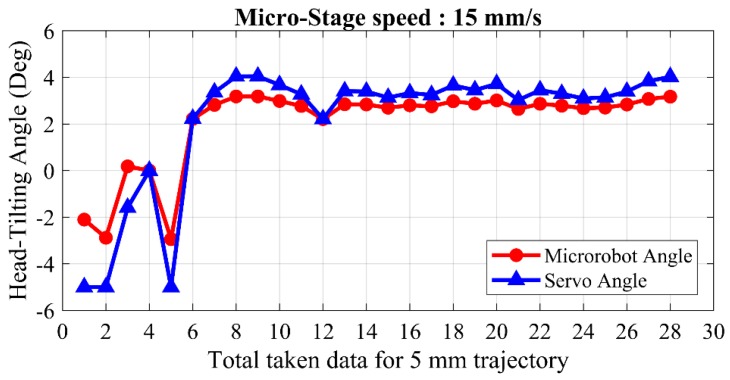
Experimental test results of the laser model controller during horizontal movement of the microrobot at a micro-stage speed of 15 mm/s.

In the next experiment, the change in head-tilting angle is investigated by running the micro-stage at a speed of 15 mm/s. Results of this experiment are given in Figure 17. The first six data points show unstable changes in head-tilting angle at the beginning of the motion. After the sixth data point, the head-tilting angle stabilizes at around 3°. The reason for the first six errors is related to the microrobot acceleration process [34]. Chattering occurs at the head-tilting angle until it reaches the desired speed. Although a faster and more stable motion profile can be obtained compared to the uncontrolled and rule-based models, the steady-state error could not be eliminated, and the head-tilting angle remains at 3°.

**Figure 18:**
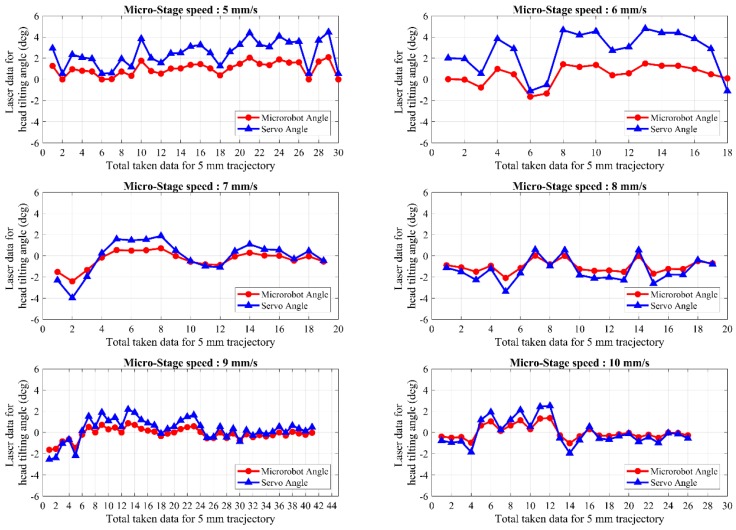
Shows the head-tilting angles of the microrobot for speeds between 5–10 mm/s with the hybrid controller.

**Figure 19:**
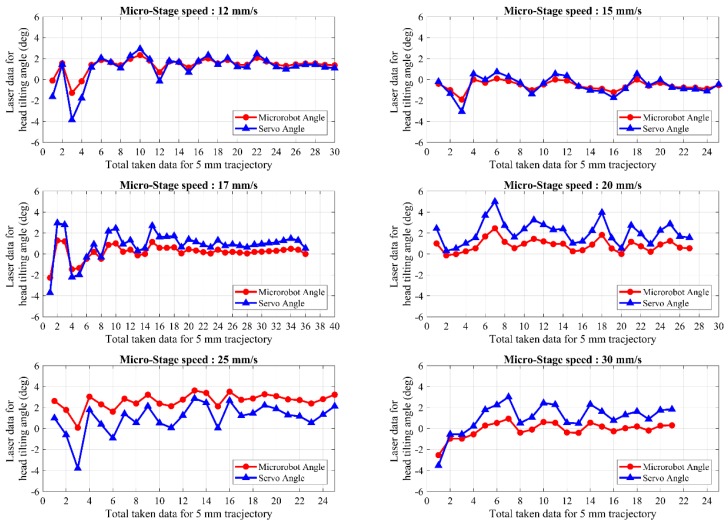
Shows the head-tilting angles of the microrobot for speeds between 12–30 mm/s with the hybrid controller.

The main reason to develop the hybrid model is the unstable change observed in the head-tilting angle during the acceleration phase when the laser model is used. A hybrid model that combines laser model and rule-based model is used to obtain better motion characteristics throughout the whole trajectory. In the experimental results given in Figure 18 and Figure 19, a trajectory of 5 mm length is successfully followed by the microrobot in a speed range of 5 mm/s to 30 mm/s. As can be seen from these figures, the unstable change that was observed for the laser model during the acceleration phase is eliminated. This enabled higher speed control to be achieved as a result of stabilization of microrobot motion. Likewise, the steady-state error is mostly eliminated, even at higher speeds. Here, the results indicate that the angle of head-tilting can be reduced independently of the speed of the motion. They also show that microrobot is capable of tracking different trajectory profiles for different applications that require high mobility, high speed, and precise localization.

**Figure 20:**
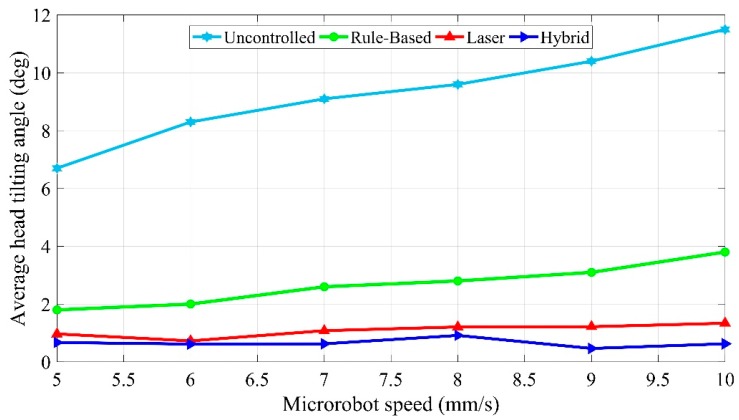
Comparison of average head-tilting angles in the speed range of 5–10 mm/s.

Average head-tilting angles for each controller for the speed range in which the experiments were conducted are given in Figure 20. As expected, high head-tilting angles that increase with microrobot speed are observed for the uncontrolled case. For the rule-based, laser, and hybrid models, ever-increasing controller performances were obtained. The average head-tilting angle values are shown in detail at Table 1 based on the comparison shown in Figure 20. As can be seen here, the head-tilting angle obtained with the hybrid model, in contrast to the uncontrolled model, is lower than 1° throughout this speed range. This average value is lower than that for the uncontrolled case, and even than the values obtained at 5 mm/s for each controller.

## 5. Conclusions and Discussion

The microrobot was intended to track a 5 mm linear trajectory at 200 μm levitation height to compare the controller performances of different models. All conducted experiments were repeated at different speeds for each control model separately, while the trajectories and levitation heights remain constant. Firstly, the microrobot movement was performed at a speed range of 5–10 mm/s in the uncontrolled model. We found that the head-tilting angle increases with respect to speed of the microrobot. At higher speeds, the robot is likely to hit the surface of the pyrolytic graphite. In experiments with the uncontrolled model, the reason for the head-tilting reaction in the *z*-axis is the drag force generated by the DI-water medium. Thus, to reduce the head-tilting angle, we developed a rule-based controller model. However, during the experimental test, it was observed that steady-state error cannot be eliminated with this model, whereas transient response can be improved. Since the rule-based model is an open-loop controller and non-linearities in the system are not taken into account for simplicity, the obtained results were unsatisfactory. Moreover, when the microrobot moves faster than 10 mm/s, the steady-state error is more likely to increase, and as a result, the microrobot head hits the surface. In the laser model, although steady state error is less than the steady-state error of the rule-based model, due to hardware limitation of sensing speed, this technique is not reliable enough for a closed-loop controller. This model can be used to control the microrobot up to speeds of 15 mm/s in stable conditions. Thus, it is not suitable for applications in which higher speeds are required. Therefore, the rule-based control system and laser calibration were merged to create a hybrid control model, resulting in a more stable control at high speeds of up to 30 mm/s. It was verified that the microrobot with the hybrid model enabled one to achieve more precise localization over a number of test results. Furthermore, in the hybrid model, the aggressiveness and the transient system response can be tuned via the rule-based model. This ensures a speed-independent, stable, closed-loop control structure and low steady-state error.

In this study, we developed various control algorithms based on discussed three models for the stabilization of the lateral motion of microrobots. There exists no prior study for the solution to this problem. By taking into consideration the complex motion tasks of microrobots in 3D space, a sinusoidal trajectory with 150 µm amplitude and 4 mm length is also tested, with an average error rate of 1.73 µm. The fact that this motion did not take place at a constant levitation height and that the microrobot has a wide range of levitation height shows the high performance of the microrobot design and orientation control. In the mathematical model section, starting from a free body diagram with head-tilting angle, the relation between the head-tilting angle and levitation height and phase difference equation depending on the micro-stage speed were derived. Thus, the mechanical delays in the controller were calculated based on the differences in the accelerations of the lifter-magnet and the microrobot. According to the calculated delay, the time required for the lifter-magnet to be held at the target orientation was determined, which in turn allowed the control of the lateral motion of the microrobot. By conducting parametric FEM analyses in COMSOL^®^, the equations that depend on micro-stage speed and microrobot head-tilting angle were determined for open-loop and closed-loop controllers. The head-tilting reaction, which can be determined using the first-order equations of angle-correction, generated undesired torque on the microrobot. It brought out, as a result, average 9.3° head-tilting angle in the case of uncontrolled model. On the other hand, by using control strategies, we achieved following results:

- 2.7° head-tilting angle for the Rule-based Model
- 1.1° head-tilting angle for the Laser Model,
- 0.7° head-tilting angle for the Hybrid Model

Moreover, the hybrid model was also experimented with at a speed of 30 mm/s and received an average angle error of 1°. This result proved that the microrobot can be used in cases in which a high speed and a complex and long trajectory are necessary. Furthermore, since the microrobot is capable of 5 axes orientation, its head position can also be utilized in various object manipulation tasks. 

As a result, through the developed control models, a solution methodology for the head-tilting problem was developed and implemented for different tasks that require high-speed and low positioning error rates. The presented control models are particularly important for the purposes of cell and particle manipulation applications. In future studies, we will focus on developing fully autonomous control techniques.

## Figures and Tables

**Table 1:** Average head-tilting angles [deg].

Speed [mm/s]	Uncontrolled	Rule-Based	Laser	Hybrid
5	6.8	1.8	0.9	0.7
6	8.3	2.0	0.7	0.6
7	9.1	2.6	1.1	0.6
8	9.6	2.8	1.2	0.9
9	10.4	3.1	1.3	0.5
10	11.5	3.8	1.2	0.6
Average (Deg, °)	9.3	2.7	1.1	0.7
